# Management of Posterior Reversible Syndrome in Preeclamptic Women

**DOI:** 10.1155/2014/928079

**Published:** 2014-11-19

**Authors:** S. Poma, M. P. Delmonte, C. Gigliuto, R. Imberti, M. Delmonte, A. Arossa, G. A. Iotti

**Affiliations:** ^1^Unit of Obstetric Anesthesia, Department of Anesthesia and Intensive Care, Fondazione Policlinico San Matteo Hospital, Viale Golgi 19, 27100 Pavia, Italy; ^2^Department of Anesthesia and Intensive Care, Fondazione Policlinico San Matteo Hospital, Viale Golgi 19, 27100 Pavia, Italy; ^3^Direzione Scientifica, Fondazione Policlinico San Matteo, Viale Golgi 19, 27100 Pavia, Italy; ^4^Dipartimento di Medicina Diagnostica e Servizi, Servizio di Radiodiagnostica, Fondazione Policlinico San Matteo, Viale Golgi 19, 27100 Pavia, Italy; ^5^Department of Obstetrics and Gynecology, Fondazione Policlinico San Matteo, Viale Golgi 19, 27100 Pavia, Italy

## Abstract

Posterior reversible encephalopathy syndrome (PRES) is a neurological syndrome associated with a number of conditions including preeclampsia. It is characterized by seizures, alteration of consciousness, visual disturbances, and symmetric white matter abnormalities, typically in the posterior parietooccipital regions of the cerebral hemispheres, at computed tomography (CT) and magnetic resonance (MRI). We report three new cases of PRES in preeclamptic patients and describe the management of these patients. We present a brief review of other cases in the literature, with particular attention to the anesthetic management.

## 1. Introduction

Posterior reversible encephalopathy syndrome (PRES) is a neurological syndrome associated with a number of conditions including preeclampsia, eclampsia, severely high blood pressure, renal failure, systemic lupus erythematosus, and the assumption of immunosuppressive agents [1, 2]. It is characterized by headache, confusion, vomiting, altered consciousness, visual disturbances, and seizures. Symmetrical white matter abnormalities suggestive of edema are seen at computer tomography (CT) and at magnetic resonance imaging (MRI), typically but not exclusively in the posterior parietooccipital regions of the cerebral hemispheres. The pathophysiology of PRES has not been completely elucidated but hypertension and endothelial injury seem to be almost always present. Vasogenic edema or vasoconstriction resulting in cytotoxic edema are probably responsible for the clinical and neuroradiological picture [3]. PRES is usually reversible, but permanent damage can occur if cerebral ischemia or hemorrhage occurs. A fast, multidisciplinary therapeutic response is thus recommended.

We present three cases of PRES in preeclamptic women, with particular attention to the anesthetic management.

## 2. Case 1

**Figure 1:**
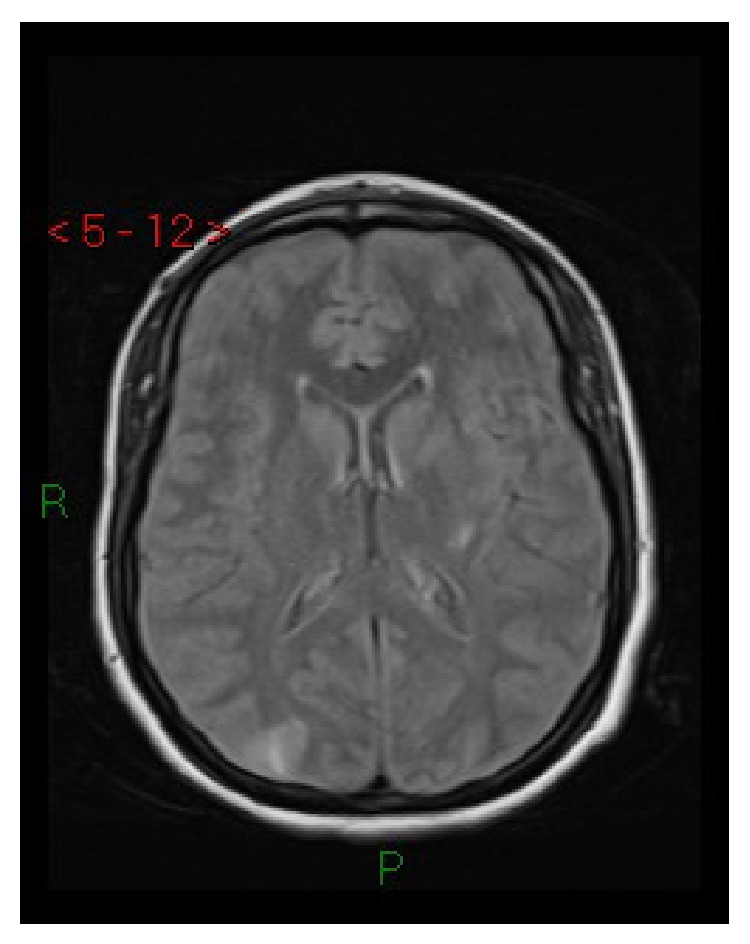
Case 1. Axial FLAIR MRI: cortical-subcortical hyperintense lesions in parietal-occipital regions and in the posterior lateral left putamen.

**Figure 2:**
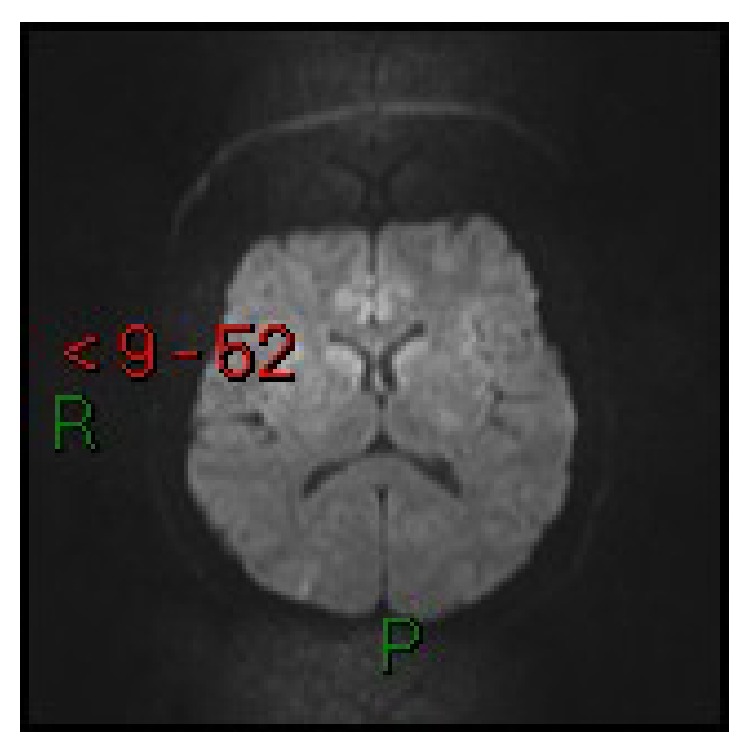
Case 1. Axial diffusion-weighted MRI: lesions do not present diffusion restriction.

A 29-year-old nulliparous African woman at 37 weeks of gestation was admitted to the obstetric emergency room with rupture of the amniotic membranes and irregular uterine contractions. Her pregnancy had been uneventful with documented normal blood pressure until 4 days before. Fifteen minutes after her arrival she appeared confused and her blood pressure was 210/120 mmHg. A state of lethargy progressively developed (Glasgow Coma Scale (GCS) 12), alternating with episodes of agitation that required sedation with intravenous midazolam. Laboratory exams showed mild elevation of LDH (410 mU/mL), AST (58 mU/mL), and ALT (47 mU/mL) with proteinuria slightly above the reference range. Partial correction of hypertension (160/110 mmHg) was achieved with oral nifedipine, intravenous labetalol, and magnesium sulphate (4 g bolus and then 1 g/h by continuous infusion). Cesarean section was performed without complications under general anesthesia. Propofol, fentanyl, and succinylcholine were used for induction. Sevoflurane 1 MAC (minimum alveolar concentration), fentanyl, and mivacurium were used for maintenance. Intraoperative monitoring was carried out with 5-lead ECG, invasive blood pressure, pulsoximetry, capnometry, and end-tidal concentration of sevoflurane. The newborn weighed 2.52 kg and presented Apgar scores of 2 at 1 minute and 6 at 5 minutes, with arterial pH 7.17 and base excess −11 millimoles/L. He was transferred to the neonatal intensive care unit. On awakening the mother was still agitated and stuporous and complained of blindness. CT showed hypodense lesions in the brain stem, in the left basal ganglia, and in the occipital lobes of the cerebral hemispheres. She was admitted to the intensive care unit (ICU). Her blood pressure was restored to normal values with oral nifedipine and intravenous continuous infusion of labetalol and urapidil. Twenty-four hours after delivery, MRI including T2-FLAIR and DWI showed a radiological picture suggestive of PRES, with several areas of altered signal intensity in the brain stem, in the inferior left temporal and occipital lobes, in the inferior right parietal-occipital lobe, and in the left basal ganglia (Figures 1 and 2), while diffusion-weighted images were normal. Forty-eight hours after delivery, neurological examination was normal, her vision had completely returned, and she was transferred to the obstetric unit. On the seventh day she was discharged from the hospital in good health with antihypertensive therapy. Follow-up MRI at 1 month from the event was completely normal. Neonatal outcome was good.

Pathological examination of the placenta revealed chronic hypoxia, decidual vasculopathy, and fibrous stroma of the chorionic villi.

## 3. Case 2

**Figure 3:**
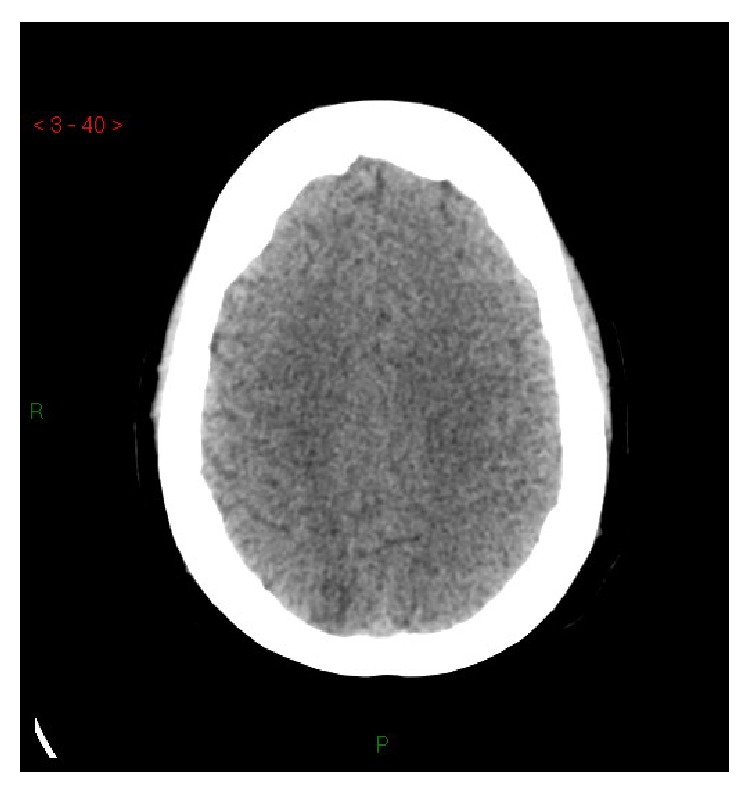
Case 2. Basal CT: hypodense cortical-subcortical lesions of the medial parietal region.

**Figure 4:**
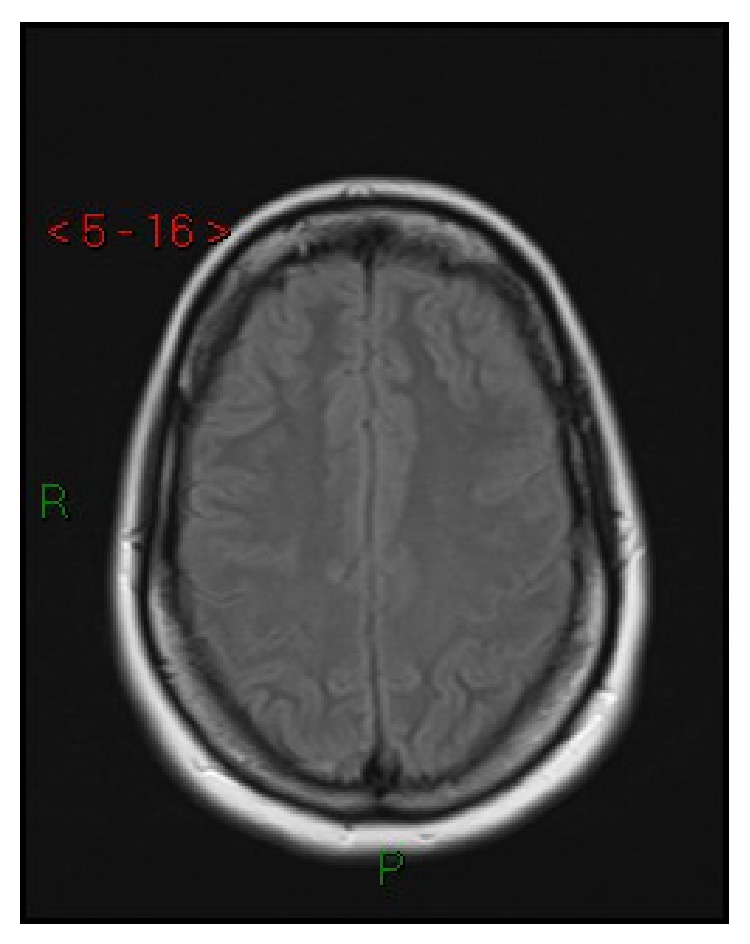
Case 2. Axial T2-FLAIR MRI: absence of lesions after 7 days.

A 26-year-old nulliparous Caucasian woman at 38 weeks of gestation came to the obstetric emergency room complaining of complete blindness and headache. She had gained 30 kilograms since the beginning of her pregnancy and weighted 120 kilograms (BMI = 44). Until then, her pregnancy had been free of complications. Her blood pressure was 205/105 mmHg and she had severe peripheral edema. Laboratory exams showed high LDH (271 mu/mL) without any other anomalies. Arterial hypertension was only partially controlled by oral nifedipine with intravenous infusion of labetalol and magnesium sulphate. Cesarean section was performed. Because of the predicted difficult management of airways (full stomach, obesity, and El Ganzouri score 6) and since she had a normal state of consciousness, single shot subarachnoid anesthesia was chosen (12 mg of hyperbaric 0,5% bupivacaine at L4-L5 level). The anesthetic sympathetic blockade, combined with the ongoing antihypertensive treatment, was associated with good control of blood pressure and no hypotension. No anesthetic or surgical complications occurred. The neonate weighed 3.8 kg and presented Apgar scores of 9 at 1 minute, and 10 at 5 minutes, with arterial pH 7.2 and base excess −6 millimoles/L. Brain CT showed hypodense occipital lesions (Figure 3). The patient was transferred to the ICU, where intravenous nitroprusside was started because of recrudescence of hypertension. Four hours after the Cesarean section, the blindness had resolved, but right facial droop and mild paresis of the right leg were observed. Pheochromocytoma and kidney dysfunction were excluded by abdominal CT and hormonal assays. Three days later she was discharged from the ICU with normal blood pressure controlled by oral antihypertensive therapy and normal neurological findings. MRI including T2 FLAIR and DWI performed 7 days later was normal (Figure 4). Diagnosis of PRES was made on the basis of the reversibility of symptoms and of the radiological lesions. On the sixteenth day she was discharged from the hospital in good health with antihypertensive therapy. Placental examination showed chronic hypoxia, intimal hyperplasia of stromal vessels, and intervillous thrombus.

## 4. Case 3

A 34-year-old nulliparous Caucasian woman at 34 weeks of gestation with just-diagnosed severe preeclampsia underwent Cesarean section under epidural anesthesia. She had presented with hypertension (190/110 mmHg), proteinuria (5 g/die), oliguria, headache, elevated ALT (475 mu/mL), AST (376 mu/mL), LDH (616 mU/mL), and intrauterine growth restriction. Her BMI was 46. She was treated with continuous infusion of magnesium sulphate and labetalol and oral nifedipine. The neonate weighed 2.09 kg and presented Apgar scores of 4 at 1 minute and 8 at 5 minutes, with arterial pH 7.18 and base excess −9.6 millimoles/L. He was transferred to neonatal ICU.

**Figure 5:**
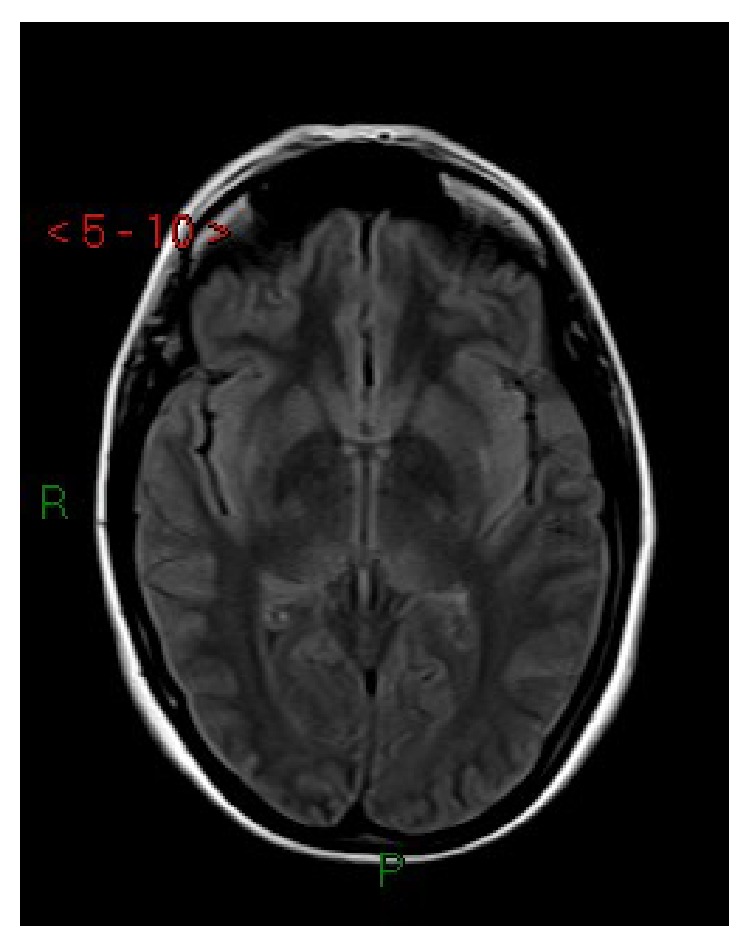
Case 3. Axial T2-FLAIR MRI: cortical-subcortical hyperintense lesions at the insular cortex and bilaterally at the lateral parts of the putamen.

**Figure 6:**
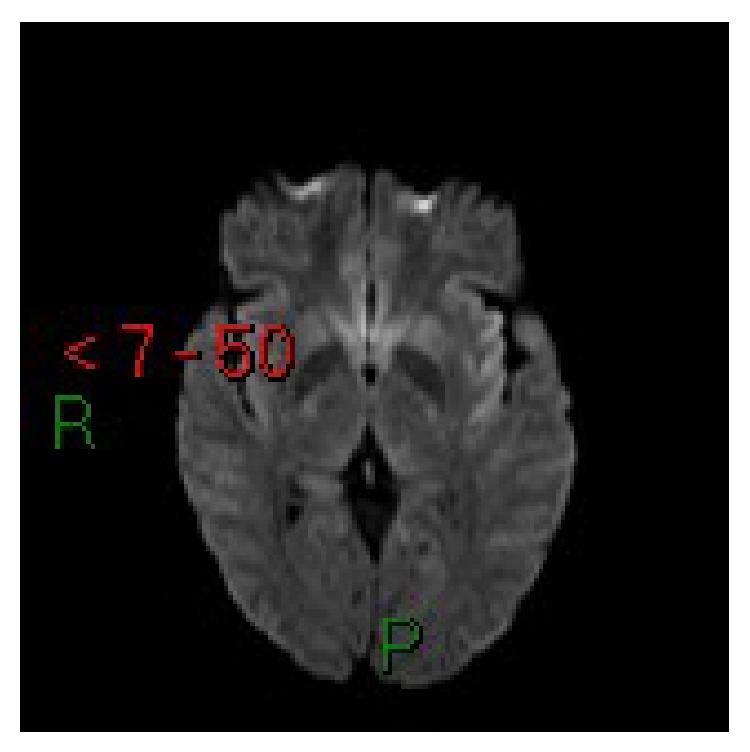
Case 3. Axial diffusion-weighted MRI: lesions showed a slight signal elevation.

Three days after delivery the magnesium sulphate and labetalol infusions were stopped because her blood pressure had stabilized and because laboratory findings had improved, but on the fourth day after cesarean section she was admitted to the ICU because of multiple generalized tonic-clonic seizures, facial droop, confusion, and inability to obey simple commands (GCS 13). Treatment with oral nifedipine and infusion of magnesium sulphate and labetalol was reinstated. Electroencephalogram showed theta and delta rhythm. Intravenous phenytoin, urapidil, and nitroprusside were introduced because of the persistence of seizures and hypertension. Pheochromocytoma and renal disease were excluded. Two CT brain scans, one at the time of admittance and another 48 hours later, were normal. However, MRI including T2-FLAIR showed altered signal intensity bilaterally in the insular cortex and putamen. The corresponding DWI showed a slight signal elevation (Figures 5-6). This finding, taken with the patient's clinical features, was suggestive of PRES. Antihypertensive therapy was continued until her discharge from ICU 8 days later, when seizures had completely ceased.

A neurological examination twenty days later was normal. She was discharged from the hospital with oral antihypertensive therapy. MRI performed 1 month later showed reduction but not disappearance of the cerebral lesions confirming that PRES is not always reversible. Placental examination showed severe chronic hypoxia, multiple infarctions, and chronic villitis-intervillitis.

## 5. Discussion

A search of the literature on PRES in obstetric patients published over the last ten years (PubMed, key words “PRES AND pregnancy,” “PRES AND preeclampsia”) turned up more than 50 reports involving 120 women, before (50%) and after (50%) delivery. PRES can be considered to be at the basis of the neurological manifestations of preeclampsia/eclampsia [4]. Some cases were observed in very early pregnancy (before the 20th week of gestation) and some were associated with intrauterine death [5]. Most women had severe preeclampsia (defined as arterial blood pressure >170/110 mmHg) but rare cases of PRES in pregnant women with normal blood pressure and without preeclampsia are also described [6].

Degree of hypertension was not associated with the extent of cerebral lesions. Cerebral edema can occur at lower levels of arterial blood pressure because of endothelium damage [7], as indicated by the fact that the most commonly reported alteration in laboratory exams is a high level of LDH [8].

The most typical symptoms in obstetric patients were seizures (45%), visual disturbances (34%, with complete blindness in 17% of these), and alteration of consciousness (19%). Focal lesions are rarely described (4%) [9]. Lesions were typically found in the posterior parietotemporooccipital areas, but also in the anterior regions (14%) [10], basal ganglia (11%) [11], stem (3%), and cerebellum (5%).

An imaging study (CT or MRI) is needed to exclude other diagnoses like cerebral venous thrombosis, or acute cerebrovascular accident, or tumor. In particular, diffusion-weighted imaging is essential to distinguish promptly between vasogenic and cytotoxic edema. Despite its superior sensitivity compared to CT (as shown in Case 3 of our study), however, MRI with diffusion-weighted imaging was performed in only 30% of the case reports [12].

Therapy is usually the same as for eclampsia: removal of the underlying cause with the performance of a Cesarean section, after a mandatory attempt of fast stabilization of the mother's status [13] by means of antihypertensive drugs, especially labetalol, nifedipine, and magnesium sulphate. The correction of hypertension must be performed cautiously, because a rapid reduction of blood pressure by more than 15%–25% can worsen the cytotoxic edema and compromise uteroplacental perfusion. Low blood levels of magnesium were found to be associated with a higher rate of radiological anomalies, while the infusion of magnesium sulphate can prevent convulsions and reduce cerebral edema [14]. The use of thiopental, valproate, or phenytoin was reported only for status epilepticus or multiple seizures and not for isolated seizures [15].

Specific cerebral antiedema therapy with steroids or hyperosmolar agents is rarely reported. Mannitol was not found to be superior to magnesium sulphate in achieving neurological recovery [16]. The outcome of pregnant women with PRES was usually reported as favorable, with resolution being rapid and complete after adequate therapy [17], although permanent damage can persist (6%), and death due to hemorrhage was described in two cases [18, 19]. Postoperatory admission of PRES patients to the ICU is advisable, to allow optimum patient monitoring and prevention of possible complications.

Although the anesthesiological management of Cesarean section has a crucial role in ensuring a positive outcome in these patients, only few papers, like ours, report information about how preeclamptic patients with PRES were approached by the anesthesiologist. In preeclamptic women, neuroaxial anesthesia is considered the best choice because it reduces systemic vascular resistance, contributing to the control of blood pressure [20]. It must thus be used unless there are contraindications. However, when neurological symptoms suggest high intracranial pressure, seizures persist, coagulopathy or thrombocytopenia is present, and general anesthesia can be required.

In Case 1, general anesthesia was chosen because of the patient's depressed state of consciousness, suggestive of cerebral damage, and because of the lack of patient cooperation. Preoxygenation followed by rapid sequence induction with propofol and succinylcholine was used to minimize the risk of aspiration. Thiopental is the agent of choice for induction in Cesarean sections, especially in the presence of eclamptic crises. Propofol has been safely used and can be considered a valid alternative to thiopental, especially in emergencies [21].

Specific attention was given to preventing hypertensive response to intubation because it is a direct cause of maternal mortality [22]. Lack of an analgesic component can increase catecholamine levels and cause blood and intracranial hypertension [23]. For this reason, fentanyl was given at the induction of anesthesia despite its ability to cross the blood placental barrier. The lower effective end-tidal concentration of sevoflurane required for an uncomplicated Cesarean section is considered to be 1.2-1.3%. This concentration prevents intraoperative awareness and minimizes negative effects on the fetus and on the uterine muscular tonus [24]. However, in preeclamptic patients with PRES, a higher concentration of sevoflurane can be used to achieve a deeper level of anesthesia with a stronger hypotensive effect, which is appropriate for this population. There are no reasons to limit the use of opioids and anesthetic vapours in these cases, because their effects on the fetus and on the uterine tone are readily reversible [25]. Magnesium sulphate can prolong the effects of nondepolarizing muscle relaxant agents. Neuromuscular monitoring is advisable when magnesium sulphate is used during general anesthesia, in order to avoid residual postoperative curarization.

In Case 2, spinal anesthesia was chosen to improve the control of hypertension and because it was considered the safest approach, given that the patient had a normal level of consciousness and was at high risk for aspiration and failed intubation. Awake fiber-optic intubation is theoretically possible but it is not practicable in emergency situations, in particular in preeclamptic women with PRES, because it can worsen hypertension and the consequent risk of cerebral complication. During neuroaxial anesthesia, the risk of hypotension, arising from sympathetic blockade, must be minimized because it can reduce maternal cerebral perfusion and lead to neurological damage. Epidural anaesthesia is preferable to spinal anaesthesia because it gives a more gradual sympathetic blockade, especially in patients treated with beta-blocking agents and vasodilatators. Epidural anesthesia also allows the best postoperative pain control because the anesthesia catheter can subsequently be used for the infusion of local anesthetics and opioids. Nevertheless, studies show that women with severe preeclampsia subjected to spinal anaesthesia experience hypotension less frequently than healthy parturients. Spinal anaesthesia can be used safely in these cases, because in preeclampsia its hemodynamic effects are similar to those of epidural anesthesia [26]. Other advantages of spinal compared to epidural anesthesia are its more rapid onset, its better intraoperative analgesic effects, and the fact that it can be carried out in the presence of moderate thrombocytopenia when epidural technique is contraindicated. Epidural tap also has a higher risk of cerebral herniation [27]. In Case 2, neither hypotension nor bradycardia occurred with the use of spinal anaesthesia despite the concomitant infusion of magnesium and of antihypertensive drugs.

Because of its vasodilatory effects, oxytocin should be preferred to methylergonovine (alpha agonist) or prostaglandins for the prevention of uterine atonia, because the latter two drugs can increase vascular resistance.

In our three cases, PRES was associated with the acute onset of preeclampsia (Cases 1 and 2) or was a complication of severe preeclampsia already under treatment (Case 3). All three women presented signs of chronic hypoxia and of impaired placentation at histology, indicating the presence of chronic disease that could have been diagnosed earlier in their pregnancy: none of the women had undergone early screening for preeclampsia despite the presence of maternal risk factors (obesity, ethnicity) [28]. Effective preeclampsia screening in the first trimester, based on a combination of clinical, biophysical, and biochemical markers, would allow the administration of therapy to improve placentation and to reduce the risk of PRES.

Our experience confirms that diffusion-weighted MRI is the most sensitive exam to confirm the diagnosis of PRES and to differentiate between reversible vasogenic and irreversible cytotoxic edema, as compared to CT scan, which can be normal in some cases of PRES (Case 3). The clinical outcomes of all three patients were good, although radiologically detectable cerebral lesions persisted in Case 3 in spite of the prompt, aggressive therapy and intensive monitoring.

Our report is one of the few in literature to describe the anesthesiological management of patients with PRES. In these patients, it is essential to limit the effects of surgical stress on hypertension and to limit the possible interaction of anesthesia with the drugs employed in the treatment of PRES.

We want to stress that the anesthetic strategies adopted for Cesarean sections on PRES patient can be different and must be adapted to the clinical status of each woman. All techniques are considered safe if performed with due respect for the specific clinical characteristics of these patients. Neuroaxial anesthesia should always be taken into consideration first because it is the least risky for mother and fetus, partly because of the antihypertensive effect of the sympathetic blockade.
